# EIF2AK3 novel mutation in a child with early-onset diabetes mellitus, a case report

**DOI:** 10.1186/s12887-019-1432-8

**Published:** 2019-03-28

**Authors:** Tarah H. Fatani

**Affiliations:** Department of Pediatrics, Section of Pediatric Endocrinology, King Abdulaziz University, Jeddah, 21589 Saudi Arabia

**Keywords:** Wolcott-Rallison syndrome, EIF2AK3 gene, Neonatal diabetes, Liver disease

## Abstract

**Background:**

Wolcott-Rallison syndrome (WRS) is caused by a biallelic mutation in the gene encoding eukaryotic translation initiation factor 2-alpha kinase 3 (EIF2AK3) on chromosome 2p11.2. This condition is characterized by permanent early-onset diabetes mellitus, epiphyseal dysplasia, and hepatic dysfunction. We report a patient with WRS born to a consanguineous marriage due to a novel biallelic frameshift mutation in the EIF2AK3 gene.

**Case presentation:**

Our patient was a 2-year-and-6-month-old Yemeni girl born to consanguineous parents who was diagnosed with neonatal diabetes at 20 days of age. She presented with chronic diarrhea and liver dysfunction. The child was normocephalic and exhibited failure to thrive and hepatomegaly with no skeletal deformities. Further investigations revealed microcytic anemia, liver impairment and primary hypothyroidism. Genetic testing confirmed the diagnosis of WRS via identification of a novel biallelic frameshift mutation in the EIF2AK3 gene. During her hospital stay, she went into septic shock and developed multi-organ failure, including fulminant hepatic failure. She unfortunately died within 2 weeks of her hospital stay.

**Conclusions:**

Wolcott-Rallison syndrome is recognized as the most common cause of early-onset diabetes in infants born to consanguineous marriages. Screening for genetic mutations in EIF2AK3 is recommended for establishing early diagnosis, providing genetic counselling, and predicting the development of additional clinical features, most importantly hepatic failure. Hence, this screening is important for guiding optimal management and improving patient outcome.

**Electronic supplementary material:**

The online version of this article (10.1186/s12887-019-1432-8) contains supplementary material, which is available to authorized users.

## Background

Wolcott-Rallison syndrome (WRS, OMIM 226980) is a rare autosomal recessive disease that was initially described in 1972 in siblings with early-onset diabetes and skeletal dysplasia [1]. The condition is characterized by permanent early-onset diabetes mellitus, epiphyseal dysplasia, hepatic dysfunction and growth retardation [2]. Additional clinical manifestations are inconsistently present and may include intellectually disability, central hypothyroidism, cardiorespiratory abnormalities, exocrine pancreatic insufficiency, renal insufficiency, neutropenia and osteoporosis [3–6].

WRS is now known to be the most common cause of early-onset diabetes in consanguineous families [7]. The condition is caused by mutations in the EIF2AK3, the gene encoding eukaryotic translation initiation factor 2-alpha kinase 3 located in the endoplasmic reticulum (ER) [8]. This enzyme, which is also known as PKR-like ER kinase (PERK), plays a key regulatory role in translation control during the unfolded protein response (UPR) in the ER to maintain cellular integrity [2]. Once activated by the accumulation of misfolded proteins during stress, PERK phosphorylates the alpha subunit of the eukaryotic initiation factor-2 (EIF2A). Upon phosphorylation, EIF2A reduces the synthesis of misfolded proteins and increases the expression of activating transcription factor 4 (ATF4) that leads to regulation of specific genes involved in autophagy, amino acid metabolism, oxidative stress and apoptosis [2, 8, 9]. PERK is highly expressed in both pancreatic beta cells and bone tissue. PERK regulates pancreatic islet cell differentiation and proliferation, proinsulin processing, folding and trafficking in pancreatic B cells and stimulates bone development [10]. Loss-of-function mutations in the EIF2AK3 gene decrease the ability of the ER to cope with stress, which results in loss of functional coordination among PERK-dependent ER chaperones responsible for controlling protein synthesis and proinsulin aggregation [11]. These effects lead to B cell defects and cell apoptosis, which result in permanent neonatal diabetes and epiphyseal dysplasia [8, 10, 11].

WRS has poor prognosis; most children with this condition die during childhood due to acute fulminant hepatitis and/or renal failure [2, 7, 12].

Here, we report a novel mutation in the EIF2AK3 gene in a 2-year-and-6-month-old girl with neonatal diabetes, pancreatic enzyme deficiency, recurrent hepatitis, primary hypothyroidism and normocytic anemia.

## Case presentation

Our patient was a 2-year-and-6-month-old Yemeni girl diagnosed with neonatal diabetes at 20 days of age on intensive insulin therapy who presented with chronic diarrhea and liver dysfunction for further evaluation. She was born at term, with a birth weight of 2000 g, and an unremarkable peinatal history, from a healthy consanguineous parent (Additional file 1: Figure S1). 

At the age of 18 months, she developed chronic diarrhea with greasy frequent stool. At the age of 22 months, she was admitted with diabetic ketoacidosis and acute liver dysfunction that resolved spontaneously. She had 3 previous hospital admissions with diabetic ketoacidosis. At the age of 2 years and 6 months, she presented at King Abdul-Aziz Hospital for the first time with a case of neonatal diabetes, chronic diarrhea with dehydration, and liver dysfunction for further evaluation and management. During her admission, she continued to have loose, greasy, pale stool. She exhibited appropriate development for her age.

Physical examination revealed that her height and weight were at the 3rd percentile; she was normocephalic. She had no facial dysmorphism and a normal eye exam. She also had hepatomegaly with a span of 10 cm with no splenomegaly. No apparent skeletal deformities were noted.

Initial laboratory tests revealed microcytic hypochromic anemia Hb 9.2 g/dl (10.9–13.8 g/dl), normal blood gas, normal kidney function, normal C-peptide levels, negative diabetes-associated autoantibodies, and uncontrolled glycemic control with an HbA1C of 11%. She had primary hypothyroidism: TSH 9.5 μIU/L (0.27–4.2 μIU/L) and FT4 13 pmol/L (12–22 pmol/L). She was administered 25 mcg of l-thyroxine daily. We investigated common causes of chronic diarrhea, but these causes were excluded. Stool fecal elastase was not tested as it is not available at our hospital. However, given the clinical picture, we highly suspected an exocrine pancreatic insufficiency. Regarding hepatic dysfunction, her liver function test revealed a high AST of 166 U/L (normal range, 15–37 U/L), ALT of 107 U/L (normal range, 12–78 U/L), total bilirubin of 3 Umol/L (normal range 0–17 Umol/L), and a normal coagulation profile. Viral infection, autoimmune hepatitis and metabolic problems were all excluded. Abdominal ultrasound revealed diffuse coarse hepatomegaly (10.6 cm) and a small hyperechoic pancreas.

The clinical diagnosis of WRS was suspected given the presence of neonatal diabetes, recurrent episodes of acute liver failure, and chronic diarrhea most likely caused by an exocrine pancreatic insufficiency. Genomic DNA was extracted from her peripheral blood sample using a standard method. A skeletal survey of the skull, ribs, spine, pelvis, long bones (humerus and femur) showed no evidence of bone anomalies.

Within less than one week following febrile illness, her clinical condition rapidly deteriorated as she developed septic shock with multisystem organ failure, including respiratory failure, hypotension, acute renal failure and fulminant liver failure with disseminated intravascular coagulation and pancytopenia. Her liver function deteriorated as follows: AST peaked to > 20,000 U/L (15–37 U/L), ALT peaked to 8944 U/L (12–78 U/L), a high total bilirubin of 140 Umol/L (0–17 Umol/L), a high direct bilirubin of 94 Umol/L, albumin was reduced to 23 g/L (40.2–47.6 g/L) and a high ammonia level of 283 Umol/L (11–32 Umol/L) with disseminated intravascular coagulation.

A multidisciplinary meeting involving the pediatric intensivist, pediatric endocrinologist, and pediatric gastroenterologist was held to determine the best management plan for this child’s complex case, and the consensus was to arrange for a transfer for a possible life-saving liver transplantation. Her parents were continually involved in the decision-making.

Needless to say, all necessary measures were implemented in our pediatric intensive care unit, including mechanical ventilation, inotropic support, renal dialysis, fresh frozen plasma, vitamin K and broad-spectrum antibiotics. Unfortunately, she went into asystole and died within 2 weeks of her admission.

Three weeks post mortem, we received the clinical exome sequencing analysis report.

Exome sequencing analysis identified that the patient harbored a novel biallelic frameshift variant (c.137_del GCCTCGGGGCGGCCGCTGCTCCCACCTCAGCGACG, p.Gly46AlafsTER19 [AHAE1] in the EIF2AK3 gene (OMIM # 60432). Genomic DNA from the specimen was enriched for the complete coding regions and splice junctions of the genes on the used panel. The products were sequenced using an Illumina Miseq instrument with 2 × 150 paired-end reads. The sequence was aligned to reference sequences based on the human genome build GRCh37/UCSC hg 19. Capillary sequencing was used to confirm relevant variants with clinical or uncertain significance. All sequence alterations were described according to the Human Genome Variation Society (HGVS). Data analysis was performed using gene-specific filtering sequencing analysis.

A follow-up appointment was arranged with the parents to disclose the genetic testing results and provide further counseling and support. Unfortunately, we couldn’t perform mutation analysis testing on the parents due to their low socioeconomic status. Furthermore, the parents are from Yemen which makes commuting to our hospital in Jeddah quite difficult.

## Discussion

We report a novel biallelic frameshift mutation p.Gly46AlafsTER19 [AHAE1] in the EIF2AK3 gene in a child with features of WRS, including neonatal diabetes, failure to thrive, recurrent hepatitis, exocrine pancreatic insufficiency, and primary hypothyroidism without any skeletal deformities. Our patient achieved appropriate developmental milestones. Previous reports on genetic testing in patients with WRS from areas with high consanguineous marriages revealed that Iranians, Arabs and the Middle Easterners harbor different homozygous variants in EIF2AK3 [3, 4, 6]. To the best of our knowledge, this biallelic frameshift variant in the EIF2AK3 gene is a novel mutation and has not been reported previously at this location.

In total, 83 WRS cases have been reported in the literature worldwide through 2013 [4]. However, WRS is currently the most common cause of PNDM in patients born from consanguineous parents [2, 7]. The clinical features of WRS might not be evident until the infant is 2 years of age. The clinical presentation and severity varies among patients, but WRS is mainly characterized by the presence of neonatal diabetes, recurrent episodes of acute liver failure, skeletal dysplasia, growth failure, exocrine pancreatic insufficiency, intellectual deficit, hypothyroidism and neutropenia [3, 8, 13]. This clinical variability does not seem to be dependent on the EIF2AK3 mutation with the possible exception of older age and neutropenia [13]. In a large international cohort study by De Franco et al., the nondiabetes features in patients with WRS were present in 12% of those referred within less than 3 months from the diabetes diagnoses but present in 83% of those with late referrals more than 48 months from the diagnosis [5].

Primary hypothyroidism was described in a child with WRS due to mutation (W521X) [14].

Here, our child had a high TSH with a low FT4 as well. The possible underlying mechanism of primary hypothyroidism might be that a deficiency in EIF2A decreases type 2 deiodinase synthesis during ER stress, leading to reduced intracellular thyroid hormone activation [15].

In the study by Habeb et al., liver disease occurred in 24 of 28 patients with WRS (85% frequency) [12]. Liver impairment is typically triggered by a viral illness, stress or hypoglycemia related to tight control of neonatal diabetes. Nonimmune hepatitis is typically recurrent with variable degrees of severity ranging from spontaneous resolution to the development of multiorgan failure and death [2, 12]. Liver failure is the most common life-threatening complication, and it may be prevented through a timely liver transplantation [12]. Our patient had multiple factors that triggered the episode of fulminant hepatic failure including the septic episode, hypoglycemia secondary to the intensive insulin therapy, and chronic diarrhea. She developed multiorgan dysfunction and ultimately died from asystole. Unfortunately, she presented to our institution later in her disease course, and we did not have a definitive diagnosis, which hindered providing her with the best anticipated management plan.

Given that WRS affects additional organs, including the pancreas and kidneys, a combined organ transplant might represent an optimal therapy that would increase survival in complicated cases. Tzakis et al. reported the first child to undergo a successful combined liver, pancreas and kidney en bloc transplantation for the management of a life-threatening complications of WRS [16]. The lack of international experience with combined organ transplant in the management of WRS and the uncertainty of the long-term prognosis, including neurocognitive outcomes, warrants further studies to assess the benefits and risks of transplantation procedures in children with life-threatening complications of WRS.

## Conclusion

Wolcott-Rallison syndrome is recognized as the most common cause of early-onset diabetes in infants born to consanguineous marriages. Screening for genetic mutations in EIF2AK3 is recommended for establishing early diagnosis, providing genetic counselling, and predicting the development of additional clinical features, most importantly hepatic failure. This information can be used to guide optimal management and improve patient outcome.

## Additional file

Additional file 1: **Figure S1.** Pedigree of the family with the proband. •Affected proband with WRS. (JPG 74 kb)

## Abbreviations

ATF4: Activating transcription factor 4
EIF2A: Eukaryotic initiation factor-2
EIF2AK3: Eukaryotic translation initiation factor 2-alpha kinase 3
ER: Endoplasmic reticulum
PERK: PKR-like ER kinase
UPR: Unfolded protein response
WRS: Wolcott-Rallison syndrome
