# Supplementary figures and images for: EIF2AK3 novel mutation in a child with early-onset diabetes mellitus, a case report
Source: BMC Pediatr. 2019 Mar 28;19:85. doi: 10.1186/s12887-019-1432-8 (PMC6438019; doi:10.1186/s12887-019-1432-8)

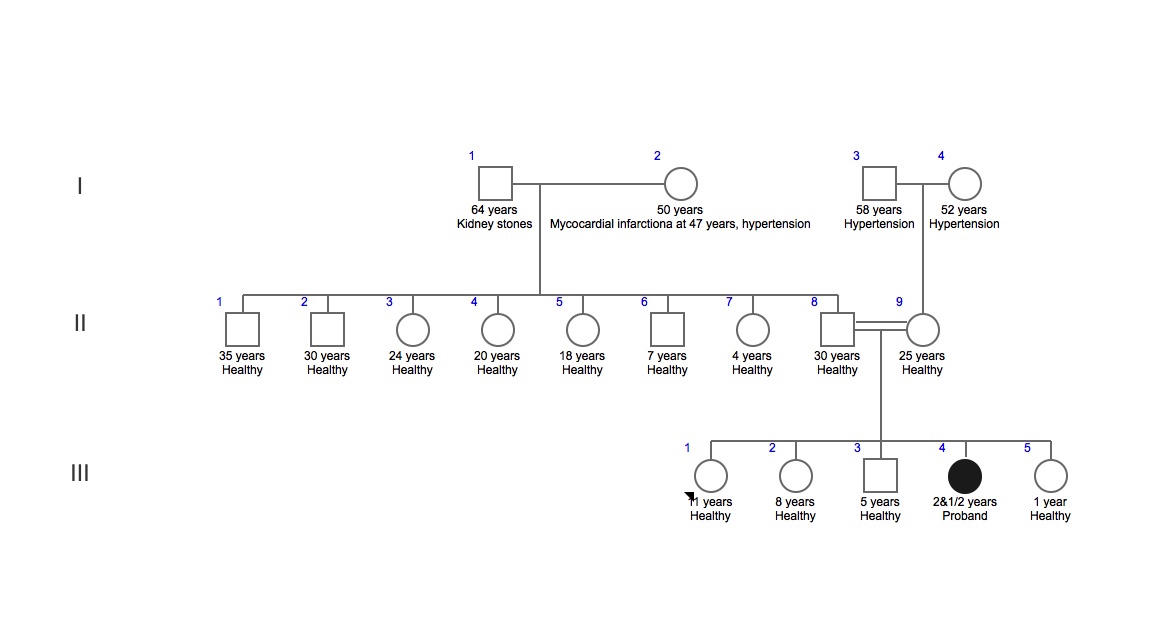

Supplement: Supplementary file 1 — Figure S1. Pedigree of the family with the proband. •Affected proband with WRS. (JPG 74 kb) [file 12887_2019_1432_MOESM1_ESM.jpg]
